# Supplementary material for: Mechanisms governing the pioneering and redistribution capabilities of the non-classical pioneer PU.1
Source: Nat Commun. 2020 Jan 21;11:402. doi: 10.1038/s41467-019-13960-2 (PMC6972792; doi:10.1038/s41467-019-13960-2)
Supplement: Supplementary file 7 — Source data [file 41467_2019_13960_MOESM7_ESM.zip › Source_Data/Figure6/Figure6B_MotifScanOutput/ETS1_PU.1/homerResults/motif36.similar.html]

motif36

## Information for motif36

C
G
T
A
T
A
C
G
C
T
G
A
A
C
T
G
C
T
A
G
T
G
C
A
C
G
T
A
T
A
C
G
T
C
G
A
C
G
T
A
T
C
A
G
T
G
C
A
C
T
G
A
C
T
A
G
  
Reverse Opposite:  

G
A
T
C
A
G
C
T
A
C
G
T
A
G
T
C
A
C
G
T
A
G
C
T
A
T
G
C
G
A
C
T
A
C
G
T
A
G
T
C
A
G
T
C
A
G
C
T
A
G
T
C
G
C
A
T
  

|  |  |
| --- | --- |
| p-value: | 1e-40 |
| log p-value: | -9.316e+01 |
| Information Content per bp: | 1.758 |
| Number of Target Sequences with motif | 327.0 |
| Percentage of Target Sequences with motif | 2.07% |
| Number of Background Sequences with motif | 206.8 |
| Percentage of Background Sequences with motif | 0.64% |
| Average Position of motif in Targets | 86.5 +/- 54.2bp |
| Average Position of motif in Background | 93.9 +/- 53.4bp |
| Strand Bias (log2 ratio + to - strand density) | -0.4 |
| Multiplicity (# of sites on avg that occur together) | 1.00 |
| Motif File: | file (matrix) reverse opposite |

### Similar de novo motifs found

|  |  |  |  |  |  |  |  |
| --- | --- | --- | --- | --- | --- | --- | --- |
| Rank | Match Score | Redundant Motif | P-value | log P-value | % of Targets | % of Background | Motif file |
| 1 | 0.880 | A C T G C T G A A C T G T A C G C G T A C G T A T C A G C T G A G T C A C T A G G T C A C G T A C T A G C G T A | 1e-24 | -56.103249 | 0.75% | 0.14% | motif file (matrix) |
| 2 | 0.777 | C T A G C T G A A C T G C T A G T G C A G T C A A T C G C T G A C G T A T A C G C T G A A C T G A C T G T G C A | 1e-23 | -54.157939 | 1.10% | 0.32% | motif file (matrix) |
| 3 | 0.812 | G T A C A C G T A C G T G T A C C G A T A G C T T G A C A C G T C G A T A G T C A C G T A G C T A T G C | 1e-21 | -48.456932 | 1.43% | 0.55% | motif file (matrix) |
